# A Sequential Machine Learning-cum-Attention Mechanism for Effective Segmentation of Brain Tumor

**DOI:** 10.3389/fonc.2022.873268

**Published:** 2022-06-01

**Authors:** Tahir Mohammad Ali, Ali Nawaz, Attique Ur Rehman, Rana Zeeshan Ahmad, Abdul Rehman Javed, Thippa Reddy Gadekallu, Chin-Ling Chen, Chih-Ming Wu

**Affiliations:** ^1^ Department of Computer Science, GULF University for Science and Technology, Mishref, Kuwait; ^2^ Department of Software Engineering, University of Sialkot, Sialkot, Pakistan; ^3^ Department of Information Technology, University of Sialkot, Sialkot, Pakistan; ^4^ Department of Cyber Security, Air University, Islamabad, Pakistan; ^5^ School of Information Technology and Engineering, Vellore Institute of Technology, Vellore, India; ^6^ School of Information Engineering, Changchun Sci-Tech University, Changchun, China; ^7^ School of Computer and Information Engineering, Xiamen University of Technology, Xiamen, China; ^8^ Department of Computer Science and Information Engineering, Chaoyang University of Technology, Taichung, Taiwan; ^9^ School of Civil Engineering and Architecture, Xiamen University of Technology, Xiamen, China

**Keywords:** VGG19, UNET, attention mechanism, brain tumor segmentation, MRI, BRATS

## Abstract

Magnetic resonance imaging is the most generally utilized imaging methodology that permits radiologists to look inside the cerebrum using radio waves and magnets for tumor identification. However, it is tedious and complex to identify the tumorous and nontumorous regions due to the complexity in the tumorous region. Therefore, reliable and automatic segmentation and prediction are necessary for the segmentation of brain tumors. This paper proposes a reliable and efficient neural network variant, i.e., an attention-based convolutional neural network for brain tumor segmentation. Specifically, an encoder part of the UNET is a pre-trained VGG19 network followed by the adjacent decoder parts with an attention gate for segmentation noise induction and a denoising mechanism for avoiding overfitting. The dataset we are using for segmentation is BRATS’20, which comprises four different MRI modalities and one target mask file. The abovementioned algorithm resulted in a dice similarity coefficient of 0.83, 0.86, and 0.90 for enhancing, core, and whole tumors, respectively.

## Introduction

Glioma is the most common type of tumor that is difficult to detect, with the lowest survival rate of 22% and constituting about 33% of all brain tumors (1–3). Some brain tumors are noncancerous, called benign, with a high survival rate, and some brain tumors are cancerous, known as malignant, with a low survival rate. There are also two types of brain tumors based on origin. The first is a primary brain tumor because it originates in the brain and occurs due to abnormal brain cells; it is also known as mutations. As cells mutate, they grow to multiply uncontrollably, forming a mass or tumor. A brain tumor is among the leading cause of death. Conversely, tumors that have spread to the brain from other locations in the body are known as brain metastasis, or secondary brain tumors (4). According to a 2019 report from the London Institute of Cancer and World Health Organization (WHO),^1^ there are approximately eighteen million registered cancer cases worldwide. Of these, 286,000 cases are brain tumors, and the highest cases of brain tumors are reported in Asia, with 156,000 cases. According to the same report, approximately 9 million deaths are due to global cancer. Out of which, 241 deaths are due to a brain tumor, and the highest mortality rate was observed in Asia with 129 cases.

Brain tumor segmentation aims to detect the extension and location of tumor regions (5). These regions are necrotic, edema, and active tissues, usually achieved by identifying abnormal areas compared to normal tissue. As glioma is the most common type of brain tumor and is hard to detect manually due to confusing boundaries, more than one MRI modality for detection was utilized (6). The different forms of MRI modalities are T1-weighted images, T2-weighted images, and fluid attenuation inversion recovery (FLAIR)-weighted images (7). These images were distinguished based on repetition time (TR) and time to echo (TE). T1-weighted images are generated using short TR and TE. T2-weighted images are generated using longer TR and TE than T1-weighted images, and FLAIR-weighted images are generated using longer TR and TE than T2-weighted images.

Previous brain tumor segmentation methods used hand-designed features. Those methods were based on the classical machine learning approach in which features are first extracted by applying statistical approaches, and then machine learning algorithms were applied for brain tumor segmentation (8, 9). In these techniques, the nature of the features did not affect the training procedure of the classifier. An alternative approach to this is automatically extracting the features used for brain tumor segmentation. This approach is most recently used and is known as deep learning. Deep learning is the study of deep neural networks (DNN), and DNN automatically learns the hierarchy of complex features directly from available data (10). Specifically, we use a pre-trained Convolutional Neural Network (CNN) (11, 12), i.e., VGG19, for brain tumor segmentation. CNN is the most widely used DNN for computer vision tasks. Similar to DNN, the standard CNN comprises the input, hidden, and output layers. The different hidden layers are convolutional, pooling, and fully connected. The working of CNN is simple: it compares the image pixels. These pixels are also known as the features of the image.

**Figure 1:**
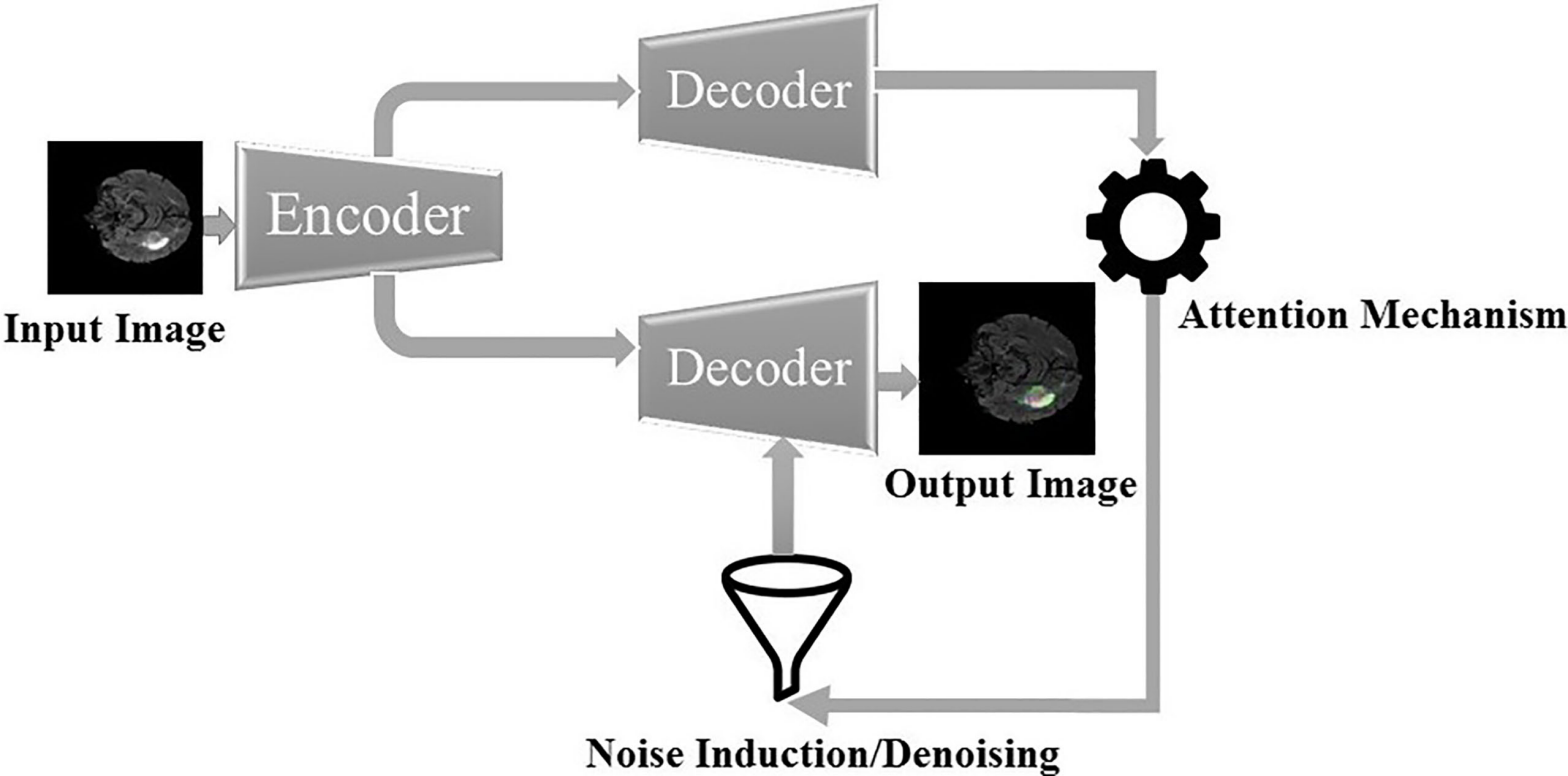
Overview of the sequential framework.

To summarize, a pre-trained CNN learned the pixels of the image by passing through different hidden layers. Therefore, in this research, we apply CNN to automatically learn feature hierarchy and utilize it for brain tumor segmentation. Subsequently, the binary classification of tumors and nontumorous regions are performed, and their results are utilized to classify all types of tumors. An overview of the whole sequential research methodology is presented in **Figure 1**. Specifically, we will propose a fully automatic, efficient encoder-decoder architecture by using BRATS’20 datasets.

The main contributions of this research article are summarized as;

- An attention-based mechanism reduces computational complexity problems and improves brain tumor segmentation results. Specifically, an image processing and attention mechanism are applied to extract the specified area of the image, followed by a pre-trained encoder part to extract the minimum but valuable features for further improving the results with efficiency.
- The implementation of the proposed framework in PYTHON using state-of-the-art libraries. The complete code is available on the GitHub repository. https://github.com/alinawazT/Brain-Tumor-Segmentation
- The validation of the proposed method was performed on the BraTS’20 and improved the Dice Similarity Coefficient (DSC) of enhancing, whole, and core tumors with 0.83, 0.90, and 0.86, respectively.

The rest of the paper is organized as follows: Section 2 highlights the previous work related to the brain tumor and addresses the research gaps. The proposed methodology is presented in Section 3. The comparison of the results with the state-of-the-art methods is presented in Section 4. Finally, Section 5 concludes the research paper with expected future research.

## Related Works

A brain tumor is hard to detect manually due to nonuniform shapes and confusing boundaries (13). Therefore, deep learning and image processing play an essential role in early brain tumor diagnosis. Different intelligent techniques were proposed for automatic early diagnosis and segmentation of the tumor region. Among them, CNN and Ensemble learning are the most widely used techniques. A short review of some of the prominent and latest techniques is presented below.

Zeldin et al. (14) apply different pre-trained deep learning architectures for fully automatic segmentation of brain tumors. Specifically, different CNN models such as dense convolutional network (DenseNet), residual neural network (ResNet), and NASNet were utilized as encoders. Like conventional U-NET, an encoder is a CNN responsible for feature extraction followed by separate decoder parts to achieve the semantic probability map. The evaluation of the proposed method was performed on BRATS’19 datasets and achieved a DSC of 0.839, 0.837, 0.839, and 0.835 on Xception, VGGNet, DenseNet, and MobileNet encoders, respectively.

Pei et al. (13) proposed a context-aware deep neural network (CANet) framework for brain tumor segmentation. In addition to U-NET’s encoder and decoder parts, it has a context encoding module that computes scaling factors of all classes. This scaling factor learns the global representation of all tumor classes. The validation of the proposed method was performed on the BRATS’19 and BRATS’20 datasets, and the evaluation metric used in the experimentation was DSC. The DSC on the test set was 0.821, 0.895, and 0.835 for enhancing tumor (ET), whole tumor (WT), and core tumor (TC), respectively.

Ghosh et al. (15) proposed a pre-trained deep learning architecture for brain tumor segmentation. The proposed architecture is similar to standard UNET except the encoder part is pre-trained VGG16, which consists of 13 convolutional layers, five pooling layers, and three fully connected layers; therefore, the decoder also has 13 convolutional layers, five upsampling layers, and three fully connected layers. The validation of the proposed method was performed on The Cancer Imaging Archive (TCIA); an evaluation was performed on different metrics such as accuracy, DSC, and intersection over union (IoU). The proposed method’s accuracy, DSC, and intersection over Union (IoU) are 0.998, 0.93, and 0.83, respectively.

Alqazzaz et al. (16) trained a variant of Segnet for brain tumor segmentation. Specifically, four different SegNets were trained on T1, Flair, T1ce, and T2-weighted images. Four SegNets are then combined, and feature extraction is performed. Finally, a Decision Tree is applied to the extracted features to generate the predicted segmentation mask of the tumor region. The datasets used in the experimentation were BRATS’17, and the evaluation metrics were precision, recall, and F-measure. They achieved an F-measure of 0.85, 0.81, and 0.79 on the whole, enhancing, and core tumors, respectively.

Karak et al. (17) proposed an encoder-decoder deep neural network for multi-class brain tumor segmentation. The proposed architecture is called TwoPath U-NET because it learns both local and global features by using local and global feature extraction paths in the down-sampling path of the deep neural network. The validation of the proposed method was performed on BRATS’19, and DSC was the evaluation metric used in the experimentation. The DSC of the proposed method was 0.76, 0.64, and 0.58 for the whole, enhancing, and core tumors, respectively.

Silva et al. (18) presented a deep multicascade fully connected neural network for brain tumor segmentation. Specifically, the proposed architecture is composed of three deep layer aggregation neural networks, i.e., basic convolutional block, convolutional block, and aggregation block. The proposed method was evaluated using BRATS’20 datasets, and the evaluation metrics used in the experimentation were DSC and Hausdorff distance. The DSC was 0.88, 0.82, and 0.79 for the whole, enhancing, and core tumors, respectively, while the Hausdorff distance was 5.32, 22.32, and 20.44 mm for whole, core, and enhanced tumors, respectively. Murugesan et al. (19) presented a multidimensional and multiresolution ensemble neural network for brain tumor segmentation and trained a traditional machine learning model for survival prediction. Specifically, an ensemble of pre-trained neural networks such as DenseNET-169, SERESNEXT-101, and SENet-154 was utilized to segment whole, core, and enhanced tumors. The segmentation map was then produced by combining the segmentation of an ensemble of pretrained deep neural networks. The datasets used in the experimentation were BRATS’19 and achieved a DSC of 0.89, 0.78, and 0.779 for the whole, core, and enhancing tumors, respectively, and survival prediction accuracy was 34%.

Specifically, the proposed architecture extracts the multistake information by combining the 3D convolutional neural network information in the residual inception block and utilizing hyperdense inception 3D UNET. Qamar et al. (20) trained a3D UNET to classify the whole, enhancing, and core tumor classes. The validation of the proposed method was performed on BRATS 2020 datasets and achieved a DSC of 0.79, 0.87, and 0.83 for enhancing, whole, and core tumors, respectively. Zhao et al. (21) performed integration of a fully connected neural network (FCNN) and conditional random field (CRF) for brain tumor segmentation. After basic preprocessing, FCNN was applied to predict the class label probability of each pixel then the prediction output was passed to the CRF-RNN for global optimization and spatial consistency of segmentation results. The validation of the proposed architecture was performed on BRATS’13, BRATS’15, and BRATS’16 datasets. The DSC of the proposed method was between 0.79 and 0.85 for the whole tumor, 0.65 and 75 for the core tumor, and 0.75 and 0.80 for the enhancing tumor, respectively.

Zhu et al. (22) presented a holistically nested neural network for brain tumor segmentation. The multiscale and multilevel hierarchical features of the brain MRI were learned by the holistically nested neural network, which is the extension of CNN to generate the prediction map of test images of brain MRI. The evaluation of the proposed method was performed on BRATS’13 datasets, and the evaluation metrics used in the experimentation were DSC and sensitivity. The results show that the presented method outperformed the previous method with DSC and sensitivity of 0.83 and 0.85, respectively. Cui et al. (23) proposed a cascaded convolutional neural network for brain tumor segmentation. The proposed architecture is composed of two subnetworks. The first network is called the tumor localization network (TCN), and it is used to detect the tumor region from an MRI scan. The second network was called as intratumor classification network (ITCN), which was used to label the defined tumorous region into subregions. The proposed architecture was validated on BRATS’15 datasets, and DSC, sensitivity, and positive predicted value (PPV) are the evaluation metrics used in the experimentation, achieving a DSC of 0.89, 077, and 0.80 for the whole, core, and enhancing tumors, respectively.

Hoseini et al. (24) proposed a Deep Convolutional Neural Network (DCNN) for brain tumor segmentation. The proposed architecture is composed of two parts. The architecture part was used to design the network model and was composed of five convolutional layers, one fully connected layer, and a softmax layer, while the second was used to optimize the learning parameters of the network during the training phase. The evaluation metric used in the experimentation was DSC and achieved a DSC of 0.9, 0.85, and 0.84 for the whole, core, and enhancing tumors on BRATS’16 datasets. Wang et al. (25) presented a Fully Connected Convolutional Neural Network for individual segmentation of WT, ET, and TC, respectively. The first step is the segmentation of WT by proposing WNet. The segmented output is used to segment ET by proposing ENet. The output was then used to segment TC by proposing CNet. The presented methods were validated on BRATS’17 and achieved a DSC of 0.78, 0.90, and 0.83 on enhancing, whole, and core tumors, respectively.

Kamnitsas et al. (26) proposed an Ensemble of Multiple Model and Architecture (EMMA) for efficient brain tumor segmentation to determine the influence of metaparameters on individual models while reducing the risk of overfitting. Specifically, the proposed architecture is the ensemble of two 3D multiscale CNNs called DeepMedic, Fully Connected Network (FCN), and UNET. The validation of the proposed architecture was performed on BRATS’17, consisting of 215 high-grade glioma (HGG) images and 75 low-grade glioma (LGG) images. The DSC of 72.9, 88.6, and 78.5 were obtained for enhancing, whole, and core tumors, respectively. Colmeiro et al. (27) proposed a fast and straightforward 3D UNET method for automatic segmentation of brain tumors. Specifically, a two-stage 3D Deep Convolutional Network was proposed. In the first step, the whole tumor was segmented from the low-resolution volume, and then the second step was the segmented delicate tissues. The proposed method was evaluated on BRATS 2017 datasets, and DSC sensitivity, specificity, and Hausdorff distance were evaluation metrics used in the experimentation. The maximum DSC, sensitivity, specificity, and Hausdorff distance mean on unseen datasets were 0.86, 0.997, and 14.0 for the whole, enhanced, and core regions, respectively. Myronenko et al. (28) proposed an automatic 3D brain tumor semantic segmentation using encoder–decoder architecture from MRI. Specifically, a variational autoencoder was used to construct input images, and a decoder was used to impose constraints on its layer. The encoder is a pre-trained ResNet, which is followed by the respective decoder. The proposed method was evaluated on BRATS 2018, and the maximum DSC values for the enhancing, whole, and core tumors were 0.82, 0.91, and 0.86, respectively. Similarly, Hausdorff distances for the enhancing, whole, and core tumors were 8.0, 10.0, and 5.9, respectively.

Hamghalam et al. (29, 30) proposed generative techniques for brain tumor segmentation. Specifically, the proposed technique uses the Cycle-Gan as an attention mechanism for improving the contrast of the tumorous region. A model is then applied to the contrasted image for final segmentation. The performance of the proposed architecture is validated on BRATS18 datasets and achieved a DSC of 0.8%, 0.6%, and 0.5%, respectively, on the WT, TC, and ET.

Most researchers focus on improving the result of segmentation while ignoring the efficiency of the task. Therefore, the prime thing in any machine learning task is to extract the minimum but valuable features. In order to tackle this problem, we will use an attention-based mechanism that will extract the useful features from the whole MRI and further utilize it for segmentation. Similarly, to reduce the algorithmic and computational complexity of the task, we will use transfer learning compared to training complete NN from scratch. This technique helps us improve the performance of the segmentation while preserving the accuracy of the task.

## Methodology

**Figure 2:**
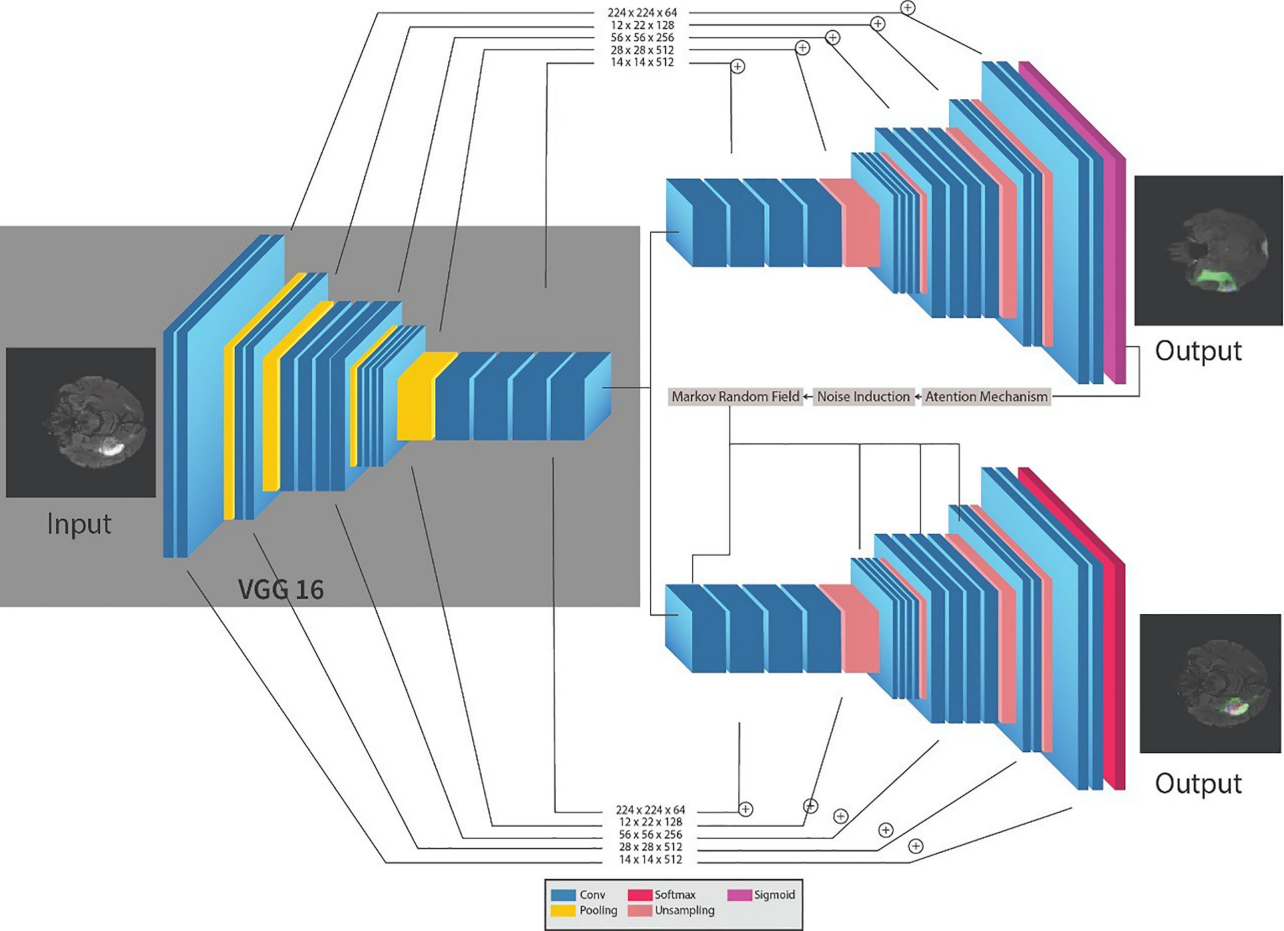
Proposed sequential framework for segmentation of brain tumor.

The framework for the segmentation of brain tumors is presented in **Figure 2**. Like the standard UNET (31) system, the proposed system contains the encoder and decoder parts. The encoder part is standard VGG19 (32)^2^ with the convolutional unit, which is used to extricate features from MR images, while the decoder part utilizes the output of the encoder VGG19 with an attention mechanism to segment the image by upsampling the element maps. The figure likewise shows that different colors address various hidden layers. The convolutional layers are represented by the blue color, the pooling layer by the yellow color, the upsampling layers by the pink color, and lastly, the SoftMax layer by the red color. Input of size (224 × 224) is given at the encoder part. In the wake of going through various hidden layers, a binary segmented image is received as the first output of the decoder part. Additionally, an attention mechanism and an overfitting reduction mechanism are applied to extract the specified segmented image and the final multiclass segmentation of the tumorous region. At first, there are 144 million boundaries of VGG19 that are diminished to 36.1 million boundaries by disposing of fully connected layers. Output is passed to the SoftMax layer to group pixels autonomously into “K” classes. K is a number of classes and is equivalent to four since we have classes with (0, 1, 2, 3) marks. 0 for nontumorous, 1 for CT, 2 for WT, and 3 for ET.

The encoder network performs convolution with a filter to produce feature maps. The rectified linear unit (ReLU) then transforms the nonlinear output into a linear output. The output is then batch normalized. Also, the max-pooling layer with 22 windows and stride 2 is performed to reduce the dimension of the image. We discard the FC layer to reduce the parameters learned from 144 to 36.1 million. The decoder, which corresponds to the initial encoder (front to the input image), generates a multichannel feature map. Similarly, the input feature map is upsampled by the decoder network. The following process represents the high-dimensional feature at the output of the last decoder. It is fed to a SoftMax classifier, which is trainable, and its output is a K channel image of probabilities. K represents the number of classes.

### Transfer Learning

Transfer learning moves the information gained by resolving one dilemma to another related issue. A model built and trained in machine learning for one dataset or recognition issue is repeatedly used as the preliminary step for the following database (33). It is difficult in practice to train a network from scratch using random initialization due to data limitations. Therefore, using pre-trained network weights as initializations or a fixed extractor of features helps solve most problems. Since pretrained models are computationally costly, it can also take a couple of days or even weeks to learn correctly from the beginning, and it also helps accelerate the training cycle, which helps in solving most of the problems at hand (25).

### VGG19

CNN is a feed-forward ANN and inspired by the human visual cortex. The neurons of CNN followed a similar connectivity pattern (2). The visual cortex is the area of the cerebral cortex that is responsible for processing visual information. Visual input is provided from the eyes and reaches the visual cortex *via* the lateral geniculate nucleus in the thalamus. The state-of-the-art artificial neural network is employed in image processing and machine vision tasks such as segmentation, classification, and recognition. The standard CNN comprises input layers, hidden layers, and output layers. The hidden layers are usually convolutional, pooling, and fully connected. The working of CNN is simple by comparing the pixels of the images (34). The pixels are also called features of the image. So, in short, CNN works by learning the features of the images, and CNN learns these features by passing through different hidden layers, and hidden layers in CNN are usually filters of different sizes. VGG19 is the commonly used CNN, composed of nineteen layers. Out of these 19 layers, 16 are convolutional layers, 5 are max-pool layers, 3 are completely connected layers, and 1 is a SoftMax layer. The architecture of VGG19 (15) is simple and follows a six-step process;

- First, the image is fed into the architecture as input; usually, the shape (224, 224, 3) is provided.
- The kernel of size (3, 3) was then applied to discover the underlying patterns of the image.
- Padding was used to preserve image resolution.
- Pooling was applied to reduce the dimension of the image.
- The output of the layers is usually linear. Therefore, a fully connected layer was applied to transform the linear output into the nonlinear output.
- Finally, the SoftMax layer is applied to predict the probability distribution of the multiple classes.

**Figure 3:**
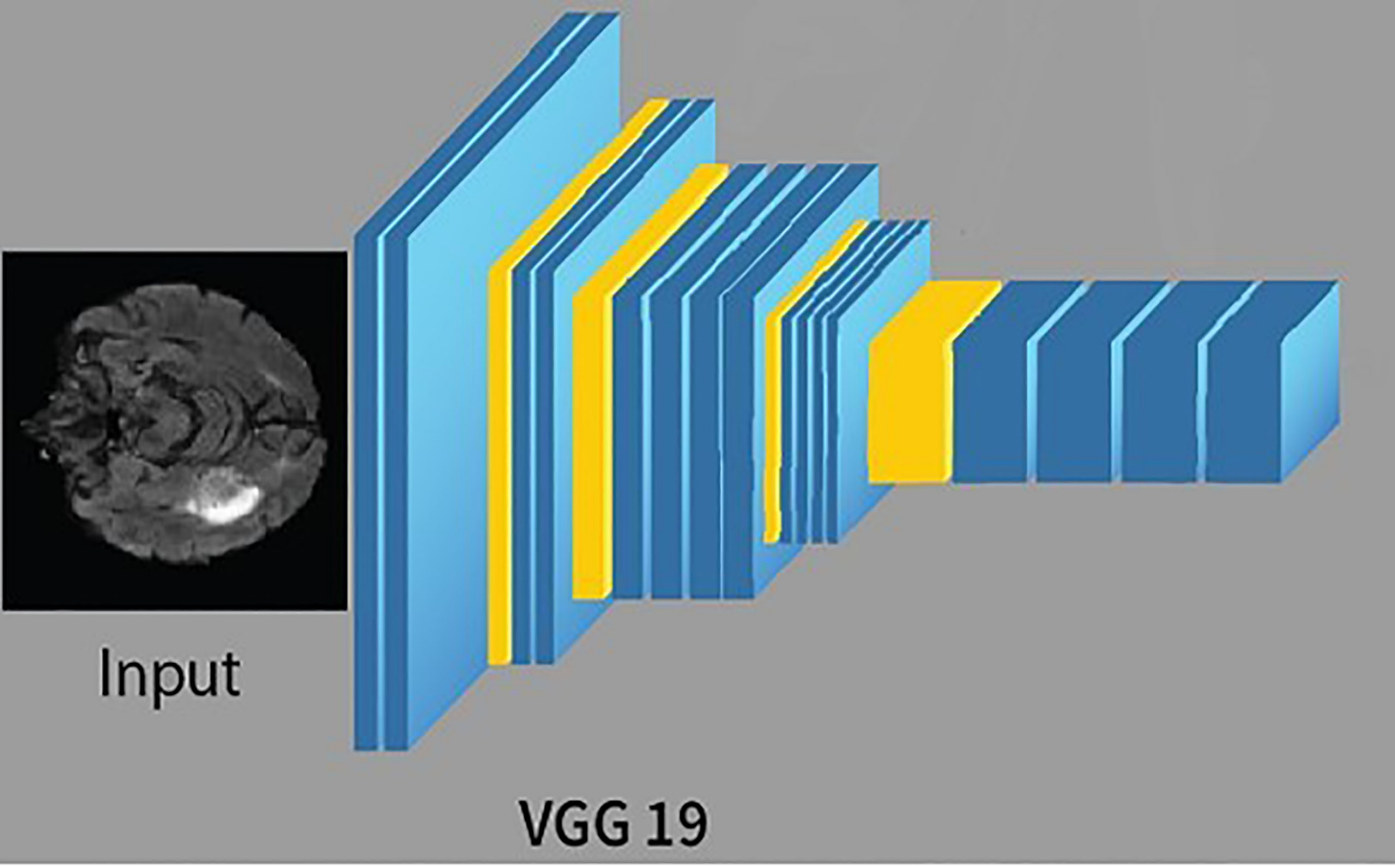
The architecture of VGG19.

The training of VGG19 from scratch is a tedious and complex task; therefore, nowadays, a pre-trained VGG19 is often used. A pre-trained VGG19 is usually trained on larger datasets, i.e., ImageNet; thus, learning new and complex patterns becomes efficient and straightforward. The architecture of VGG19 is shown in **Figure 3**.

### Attention Mechanism

The attention mechanism is a model for medical image examination that naturally figures out how to focus on the targeted image of changing shapes and sizes (35–40). An attention mechanism helps the decoder focus on the area of interest. Subsequently, with the attention mechanism, we will classify the pixel by the hidden state of the decoder. Hence, we partition the image into *n* parts; then, at that point, at the *i*th area of the image, we utilize the hidden region of the decoder part. The hidden region is then utilized as the setting to choose the interest area of the image. The *zi* is the output of the attention mechanism. Models prepared with attention mechanisms certainly figure out how to smother unessential regions in an input image while featuring remarkable features helpful for a particular task, which empowers us to eliminate the need to utilize express outside tissue/organ localization modules when using CNN. **Table 1** describes the important symbols and variables used in the equation. The mathematical representation of the attention mechanism with overfitting reduction is as follows.

**Table 1:** Symbols with description.

**Serial number**	**Symbol**	**Description**
1.	*f_A_*	Feed-forward neural network
2.	$V_{A}^{T}$	Transformation function
3.	*W_A_*	Attention function
4.	*C_t_*	Context vector
5.	*η*	Learning rate
6.	*ð*	Standard deviation

Algorithm 1: Pseudocode for the proposed approach.

1. *i*_*jt*_=*f*_*A*_(*o*_*t* −1_,*e*_*j*_) 

2. $f_{A}=V_{A}^{T}*tanh\left(U_{A}*i_{j}+W_{A}*o_{t}\right)$

3. $C_{t}=\sum_{j=1}^{T}alpha_{\mathrm{ji}}*e_{j}\;such\;that\sum_{j=1}^{T_{x}}\alpha_{jt}=1$

4. where *α*_*ij*_≧0 

5. *o*_*t*_=*CNN*(*s*_*t* =1_.[*e*(*y*_*t* =1_)·*c*_*t*_]) 

6. *A*(*x*,*y*)=*o*_*t*_+*N*(*x*,*y*) 

7 where $\text{N}\left(\text{x,y}\right)\;=\frac{1}{\sigma \sqrt{2\pi }}e^{\frac{-{(}_{z}-)}{2{ð}^{2}}}$

8. select the pixels with the lowest energy $-E_{\mathrm{total}}\left(x,y\right)=\sum_{i,j}^{N}x_{i}{,}_{j}\cdot x_{i}{+}_{1}{,}_{j}{+}_{1}-\eta \sum_{i,j=1}^{N}x_{i}{,}_{j}\cdot y_{i}{,}_{j}$

- In the algorithm, *i_j t_* represents the location of *j*th pixel in the input image
- *o_t_* is the is the output of the decoder part
- *e_j_* is the current state of the encoder part

For a linear transformation of input, a simple feed-forward neural network *f_A_* is applied and then a nonlinearity (*tanh*) and transformation function $V_{A}^{T}$ (scalar quantity). The fourth line of the pseudocode shows that, as now, we know the input, we need to feed the weighted sum combination of input to the decoder. In the following line,

- *e*(*y*_*t*−1_) is the previously predicted label of binary classification
- *C_t_* is the context vector, i.e., the weighted sum of the input

After that, noise *N*(*x*,*y*) is combined with the output of decoder *o_t_* where *N*(*x*,*y*) is the Gaussian noise function. Finally, we apply the MRF by looping over the pixels of image *A*(*x*,*y*) and computing the energies of the current pixels of *A*(*x*,*y*) by applying the formula given in the eighth line.

### Markov Random Field

The initial outcomes of the proposed models result in overfitting. Therefore, we introduce noise in the generated image of the decoder. Specifically, we used the Gaussian noise function to introduce noise. We add 20% noise to the image. The MRF algorithm is then applied to denoising the resultant image. Compared to the Bayesian network, the connection between nodes in MRF are undirected and cyclic. The MRF is defined in terms of energy. When pixels of both images match, we say that the energy of both images is low and high otherwise. The algorithm moves on to the pixels, either moving through them in some predetermined order or choosing a random pixel at each step, running through the set of pixels until their values stop changing. The equation in line 15 represents the energy of the where *η* and *ζ* are positive constant, energy of the “output pixels” and here we set *η*=15, *ð*=1.5 .

A reliable and efficient variant of a pre-trained neural network, i.e., an attention-based recurrent convolutional neural network for brain tumor segmentation, is proposed in the proposed framework. Specifically, the encoder part of the UNET is a pre-trained recurrent VGG19 network followed by the adjacent recurrent decoder part with an attention gate.

## Results

### Evaluation Metrics

In this section, the qualitative and quantitative results of the proposed framework are presented along with evaluation metrics.

Generally, two types of segmentation of brain tumors are used, i.e., manual segmentation and automatic segmentation. Firstly, the manual segmentation is performed by MRI experts, which is a tedious and complex task, but accurate, while the accurate and straightforward software does automatic segmentation due to developments in artificial intelligence. It is also worth mentioning that the MRI experts first label the datasets used for automatic segmentation (41). The evaluation metrics used for brain tumor segmentation are DSC, accuracy, sensitivity, and precision (42). Similarly, true negative (TN) refers to the negative tuple correctly labeled by the classifier. False negative (FN) refers to the classifier’s tuple to the positive tuple incorrectly labeled. Similarly, false positive (FP) refers to the negative tuple incorrectly labeled by the classifier.

**Figure 4:**
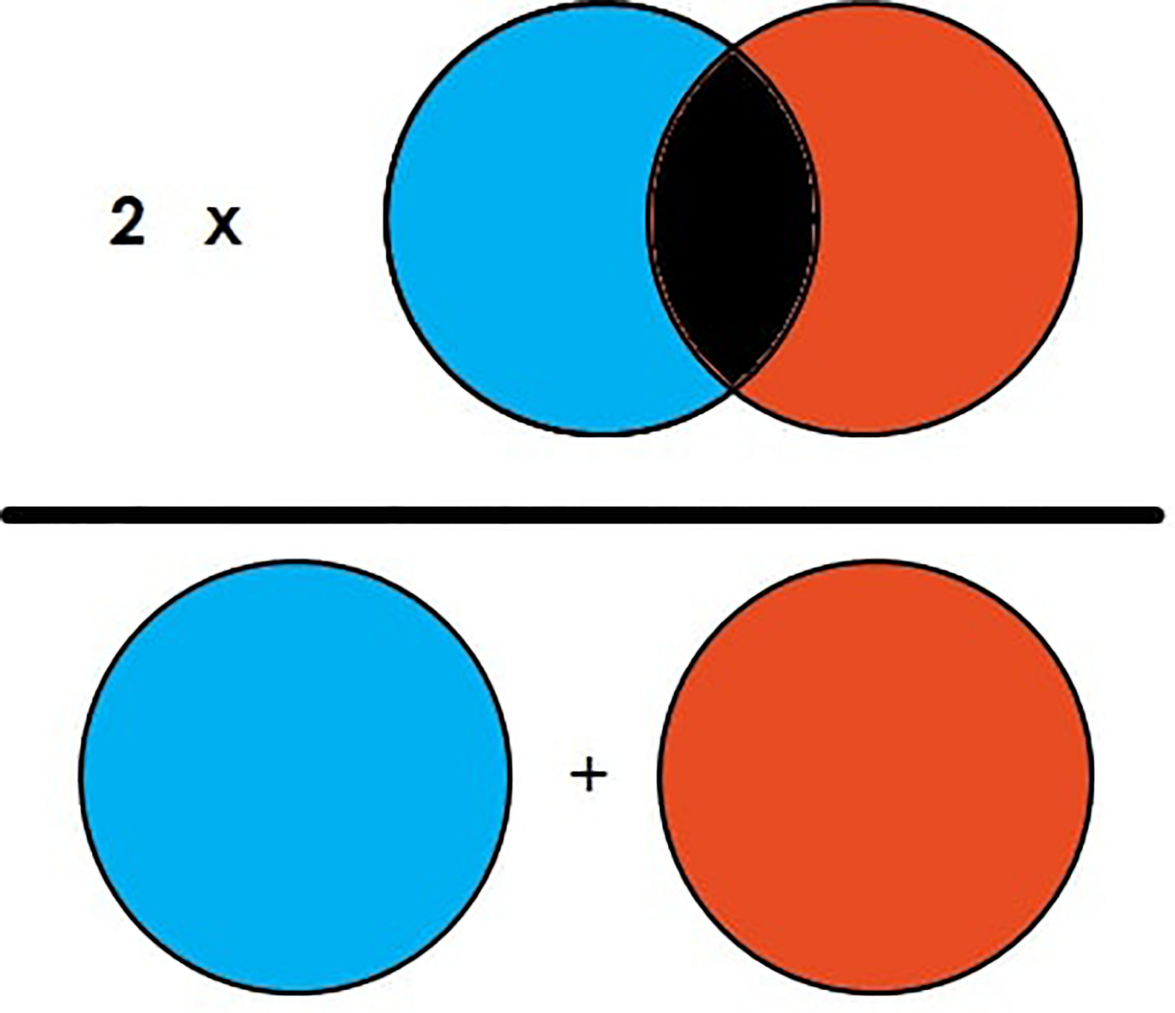
Illustration of Dice Similarity Coefficient (DSC).

DSC is the commonly used evaluation metric for image segmentation and segmentation of brain tumors. DSC is the measure of overlapping area between two images (23). For example, in **Figure 4**, there are two circle images labeled “A” and “B.” The DSC of the figure is then illustrated in **Equation 1**, which shows that DSC is equal to two times the overlapped area in the general area of the image element of both images. It can also be illustrated as two times the true positive (TP) divided by total TP, FP, and FN as represented in **Equation 2**.


(1) DSC=2|A∩B|/(|A|+|B|)

(2) DSC=2TP/2TP+FN+FP

### Qualitative Results

For the evaluation of the segmentation task, the BRATS’20 (43) was used, consisting of 371 image files, and each file is composed of five subfiles, out of which four files are MRI modalities of the individual patients, and one file is the target mask of the individual patient. T1, T2, T2*, and attenuated inversion recovery (FLAIR)-weighted mages are the most common modalities of MRI utilized in this dataset. A different clinical protocol was acquired for each modality, and multiple scanners from several institutions and each modality have been segmented manually by one to four raters. All the modalities are available as NIFTI files with the extension (.nii.gz). A NIFTI file is the most common file format for neuroimaging. The available datasets are imbalanced; therefore, in the data preprocessing step, a patch-wise training procedure is applied (44).

**Figure 5:**
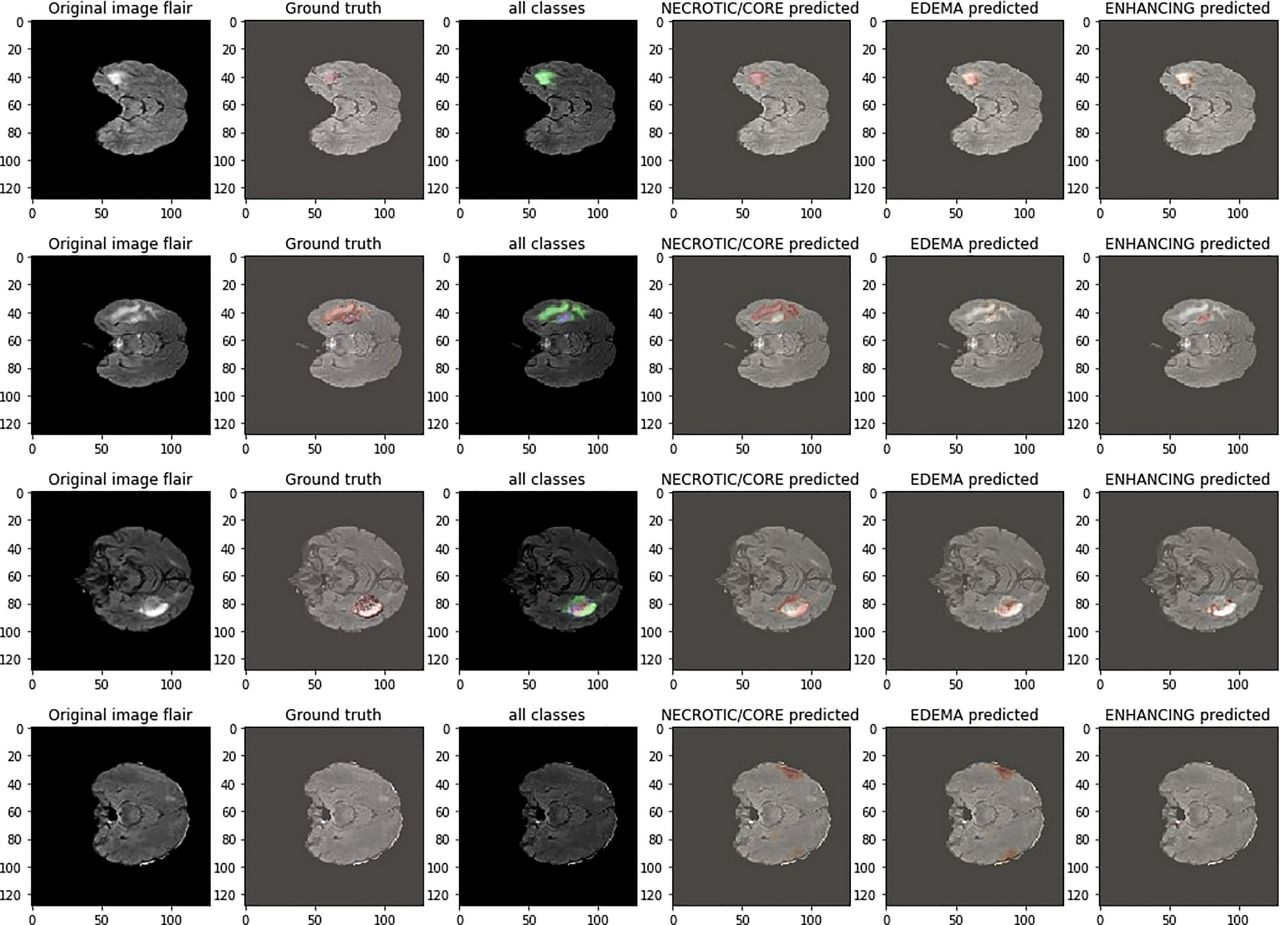
Qualitative results of the proposed framework.

**Figure 5** shows the segmentation results of the proposed model on the BRATS’20 dataset. The first column is the tumor segmentation of all tumor classes, followed by the individual segmentation of core, whole, and enhancing tumors in columns fourth, fifth, and sixth, respectively.

### Quantitative Results

The quantitative results of the proposed model are presented in **Table 2** with a sensitivity, specificity, accuracy, and precision of 0.98, 0.981, 0.99, and 0.993, respectively. Similarly, the comparison of the achieved results with the primary method is presented in **Table 3**, which reveals that the proposed framework outperformed the state-of-the-art methods. The comparison is performed based on the DSC score of ET, WT, and TC, respectively.

**Table 2:** Quantitative results of the proposed model.

Metrics	Results
Sensitivity	0.98
Specificity	0.981
Precision	0.993
Accuracy	0.99
DSC of ET	0.861
DSC of WT	0.90
DSC of TC	0.83

**Table 3:** Comparison of results of brain tumor segmentation.

Methods	ET	WT	TC
Ghaffari et al. (45)	0.78	0.90	0.82
Ballester et al. (46)	0.67	0.85	0.78
Colman et al. (27)	0.75	0.86	0.79
Proposed method	0.83	0.90	0.86

## Conclusion

In conclusion, a pre-trained VGG19 neural network with an attention mechanism and an image processing technique is trained for brain tumor segmentation. Applying the attention mechanism aims to suppress irrelevant regions in an input image while highlighting essential features useful for a specific task. The proposed model’s evaluation is carried out on BRATS20, and evaluation metrics used in the segmentation method are accuracy, sensitivity, specificity, precision, and DSC. The obtained results show that the proposed model produces more accurate and better outputs than the previous method for enhancing, whole, and core tumors with dice similarity coefficient scores of 0.83, 0.9, and 0.86, respectively. The proposed segmentation methods enable the efficient and effective diagnosis of brain tumors. In the future, an ensemble attention mechanism will be proposed to extract the more important features and increase the segmentation results.

## Data Availability Statement

The original contributions presented in the study are included in the article/supplementary material. Further inquiries can be directed to the corresponding authors.

## Author Contributions

This research specifies below the individual contributions. Conceptualization: TA and AN. Data curation: AN. Formal analysis: AJ. Funding acquisition: TA. Investigation: AJ and AR. Methodology: AJ. Project administration: C-LC, RA, and TA. Resources: RA, TG, and TA. Software: AJ. Supervision: C-LC, AJ, C-LC, and C-MW. Validation: AN, RA, and TG. Visualization: TG and C-LC. Writing—review and editing: AR, C-MW, TA, AR, and C-MW.

## Conflict of Interest

The authors declare that the research was conducted in the absence of any commercial or financial relationships that could be construed as a potential conflict of interest.

## Publisher’s Note

All claims expressed in this article are solely those of the authors and do not necessarily represent those of their affiliated organizations, or those of the publisher, the editors and the reviewers. Any product that may be evaluated in this article, or claim that may be made by its manufacturer, is not guaranteed or endorsed by the publisher.

## Footnotes

1
https://www.who.int/news-room/fact-sheets/detail/cancer

2
https://neurohive.io/en/popular-networks/vgg19/
